# Nitric oxide-releasing PHEMA/polysilsesquioxane photocrosslinked hybrids

**DOI:** 10.1039/d5ra07870a

**Published:** 2025-11-18

**Authors:** Herllan Vieira de Almeida, Laura Caetano Escobar da Silva, Bruno de Almeida Piscelli, Beatriz Rafaelle Goes dos Santos, Daniele Mendes Guizoni, Ana Paula Davel, Rodrigo Antonio Cormanich, Marcelo Ganzarolli de Oliveira

**Affiliations:** a Institute of Chemistry, University of Campinas, UNICAMP Campinas 13083-970 SP Brazil mgo@unicamp.br; b Institute of Biology, University of Campinas, UNICAMP Campinas 13083-862 SP Brazil anadavel@unicamp.br

## Abstract

Polymeric materials capable of releasing nitric oxide (NO) locally have potential uses in various biomedical applications. One of the main challenges in this field is obtaining materials that allow the modulation of NO release rates through the incorporation of different NO donor molecules. Herein, we describe the synthesis of materials composed of poly(2-hydroxyethyl methacrylate) (PHEMA) crosslinked by a polysilsesquioxane (PSS) network through sol–gel polymerization and photocrosslinking. Increasing the PSS content from 5 to 20 wt% led to an increase in the glass transition temperature from 107 °C to 133 °C. Swelling studies in phosphate buffer saline solution and ethanol revealed that higher siloxane content reduced the solvent uptake of the hybrids, while surface contact angle measurements confirmed that all compositions remained hydrophilic (60–70°). These hybrids enabled, for the first time, the incorporation of two structurally distinct NO donors, hydrophilic *S*-nitrosoglutathione (GSNO) and hydrophobic *S*-nitroso-*N*-acetyl-dl-penicillamine (SNAP), *via* absorption from aqueous and ethanolic solutions, respectively. Computational modeling showed that GSNO forms multiple hydrogen bonds with PHEMA hydroxyl groups, while SNAP interacts hydrophobically with its methyl groups. Real-time NO measurements showed that SNAP spontaneously releases NO at a flow rate 2 to 10 times higher than that of GSNO in the first 30 min after hydration of the hybrids, likely due to its weaker intermolecular interactions and higher mobility upon hydration. The hydrophilic nature of the hybrids, tunable NO release, and lack of cytotoxicity toward cultured endothelial cells position them as promising candidates for the manufacturing of antithrombotic blood-contacting medical devices.

## Abstract 2

PHEMA/PSS hybrids loaded with GSNO or SNAP release NO upon hydration. Their distinct real-time NO release profiles reflect different matrix interactions, highlighting their potential use as tunable platforms for controlled NO delivery.

## Graphical abstract

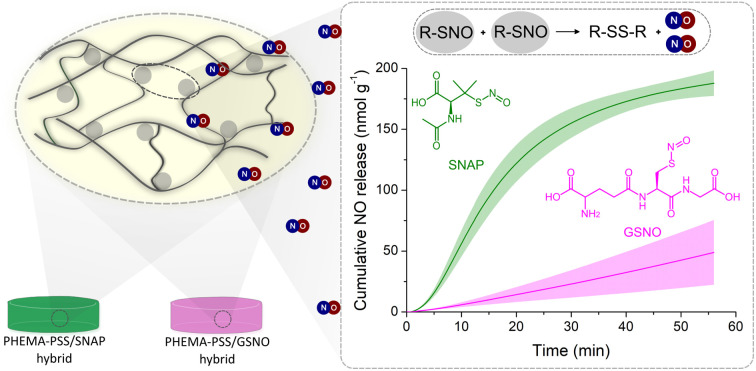

## 1. Introduction

The growing demand for advanced biomaterials in modern medicine has spurred interest in organic–inorganic hybrid systems.^1^ These materials combine organic components, such as polymers, with inorganic constituents like metals, oxides, or ceramics, offering synergistic properties that surpass those of their individual parts.^2^ While organic polymers provide flexibility and ease of processing, they often lack mechanical and thermal stability. In contrast, inorganic components offer strength and durability but are typically brittle and less versatile.^3,4^ Hybrid materials bridge this gap by integrating the advantages of both domains, allowing for precise control over mechanical, thermal, and functional properties for diverse applications.^5^

Polysilsesquioxanes (PSSs) are a prominent class of hybrid materials with the general formula (RSiO_1.5_)_*n*_, where R denotes an organic group or hydrogen. They are synthesized through the hydrolysis and condensation of R–SiX_3_ precursors (X = Cl or OR′), forming siloxane (Si–O–Si) networks with various nanostructures depending on synthesis conditions.^6,7^ The rigid inorganic backbone provides thermal stability, while the organic R groups introduce flexibility and functionalization potential.^8^ Due to their biocompatibility and inert siloxane framework, PSSs have been explored in applications such as drug delivery,^9^ photodynamic therapy,^10^ and tissue engineering.^11^

Among organic polymers, poly(2-hydroxyethyl methacrylate) (PHEMA) is widely used in biomedical materials. Derived from 2-hydroxyethyl methacrylate (HEMA), it is hydrophilic and biocompatible.^12,13^ These properties make PHEMA suitable for applications including contact lenses,^14^ catheters,^15^ and scaffolds for tissue engineering.^16^ Combining PHEMA and PSS networks^17^ offers a promising approach for creating hybrid materials with tuneable architecture and enhanced functionality for medical use.

Nitric oxide (NO) is a biologically active gas involved in key physiological processes such as vascular tone regulation,^18^ inhibition of platelet adhesion,^19^ neurotransmission,^20^ immune response modulation,^21^ and wound healing.^22^ Due to its broad biological roles, there is significant interest in biomaterials that enable localized, sustained NO delivery.^23–25^ Since NO is gaseous and reactive, it is typically introduced *via* donor molecules or functional groups capable of releasing NO under thermal or photochemical stimuli.^26^ Common NO donors include *S*-nitrosothiols (RSNOs),^27^*N*-diazeniumdiolates (NONOates),^28,29^ and metal–NO complexes.^30^

Despite growing research on NO-releasing biomaterials, few studies have focused on PHEMA- or PSS-based systems. Halpenny *et al.* developed a ruthenium–NO complex copolymerized with HEMA for UV-triggered NO release,^31^ followed by further studies using manganese–NO complexes in PHEMA hydrogels with bactericidal effects.^32,33^ Gao *et al.* created NO-releasing polymeric micelles from PHEMA functionalized with organic nitrates, achieving tuneable NO release profiles.^34^

In PSS-based platforms, Besson *et al.* functionalized aminosilane-derived PSS with NONOate groups for NO release under physiological conditions.^35^ Naghavi *et al.* developed a PSS/polyurethane hybrid impregnated with *S*-nitroso-*N*-acetylpenicillamine (SNAP) for cardiovascular grafts.^36^ Our group previously reported supramolecular foams composed of cellulose nanocrystals, PSS, and polyethylene glycol, where *S*-nitrosated thiol-functionalized siloxanes enabled spontaneous NO release upon hydration.^37^ While both PHEMA and PSS have shown promise individually for NO delivery, no studies have combined them into a single hybrid system for this purpose.

**Fig. 1:**
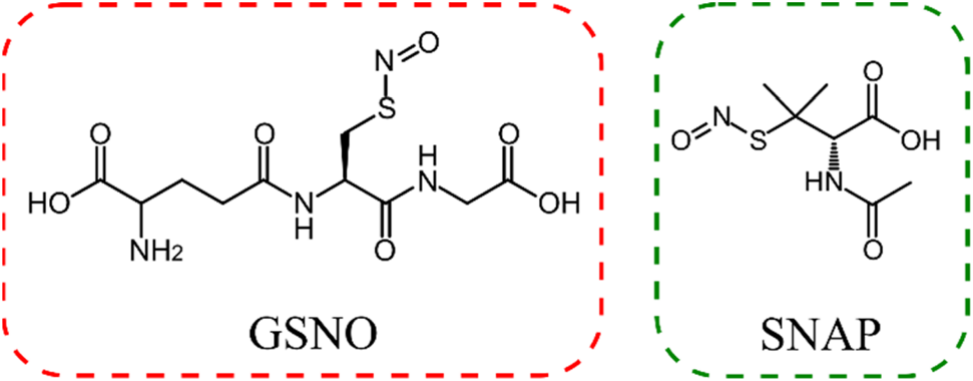
Chemical structures of *S*-nitrosogutathione (GSNO) and *S*-nitroso-*N*-acetyl-d,l-penicillamine (SNAP).

In this study, we report the synthesis and characterization of photocrosslinked PHEMA–PSS hybrids capable of releasing NO under hydration. These materials incorporate two different NO donors: the hydrophilic *S*-nitrosoglutathione (GSNO) and the hydrophobic *S*-nitroso-d,l-penicillamine (SNAP) (Fig. 1). Molecular modelling suggests that GSNO exhibits stronger interactions with PHEMA *via* hydrogen bonding than SNAP. These different intermolecular interactions lead to different NO release kinetics and holds promise for applications of NO-releasing PHEMA–PSS hybrids in medical devices.

## 2. Materials and methods

### 2.1. Materials

3-Isocyanatopropyltriethoxysilane (IPTES), 2-hydroxyethyl methacrylate (HEMA), phenylbis(2,4,6-trimethylbenzoyl)phosphine oxide (Irgacure® 819), glutathione (GSH), *N*-acetyl-dl-penicillamine (NAP), sodium nitrite (NaNO_2_), sodium chloride (NaCl), potassium chloride (KCl), sodium iodide (NaI), monobasic sodium phosphate (NaH_2_PO_4_), dibasic sodium phosphate (Na_2_HPO_4_), ascorbic acid, phosphate-buffered saline (PBS), MTT, and DMSO were purchased from Sigma-Aldrich (USA). Hydrochloric acid (37 wt%), sulphuric acid, sodium hydroxide, ethylenediaminetetraacetic acid (EDTA), methanol, ethanol, and acetone were obtained from Synth (Brazil). Cell culture reagents included human umbilical vein endothelial cells (HUVECs; Lonza #CC 2519), Endothelial Growth Medium 2 (EBM 2; Lonza #CC 3162), Hank's Balanced Salt Solution (HBSS; Sigma-Aldrich #H6648), trypsin EDTA 0.25% (Gibco #25300054), Dulbecco's Modified Eagle Medium (DMEM; Vitrocell #2325), and fetal bovine serum (FBS; Vitrocell #S0011). All chemicals were of analytical grade and used as received. Ultrapure water (resistivity 18.2 MΩ cm) was used for all aqueous solutions.

### 2.2. Synthesis of HEMA-TES and preparation of the PHEMA–PSS hybrids

The synthesis of the hybrid precursor HEMA-TES was carried out by conjugating 2-hydroxyethyl methacrylate (HEMA) with 3-isocyanatopropyltriethoxysilane (IPTES) using a solvent-free method adapted from da Silva *et al.*^38^ In brief, 4.50 g of IPTES and 2.96 g of HEMA were placed in separate round-bottom flasks, each purged with nitrogen gas for approximately 30 min to remove oxygen and moisture. The flasks were sealed with rubber septa. HEMA was then transferred to the IPTES flask using a syringe, and the reaction mixture was stirred at 70 °C under a nitrogen atmosphere. Reaction progress was monitored by periodic sampling and analysis by Fourier transform infrared (FTIR) spectroscopy to confirm the formation of HEMA-TES.

Once synthesized, the HEMA-TES was transferred to a clean round-bottom flask and mixed with additional HEMA. The mixture was stirred at 70 °C, followed by the addition of an aqueous 2 wt% HCl solution to catalyse the hydrolysis and condensation of the triethoxysilane groups. This acid-catalysed sol–gel reaction led to the *in situ* formation of the polysilsesquioxane (PSS) network. Ethanol evolution during the reaction was evidenced by bubbling, which continued for approximately 40 min. Stirring was maintained until bubbling ceased, indicating completion of the condensation step. The system was then cooled to room temperature, and the photoinitiator Irgacure® 819 was added to initiate crosslinking.

**Fig. 2:**
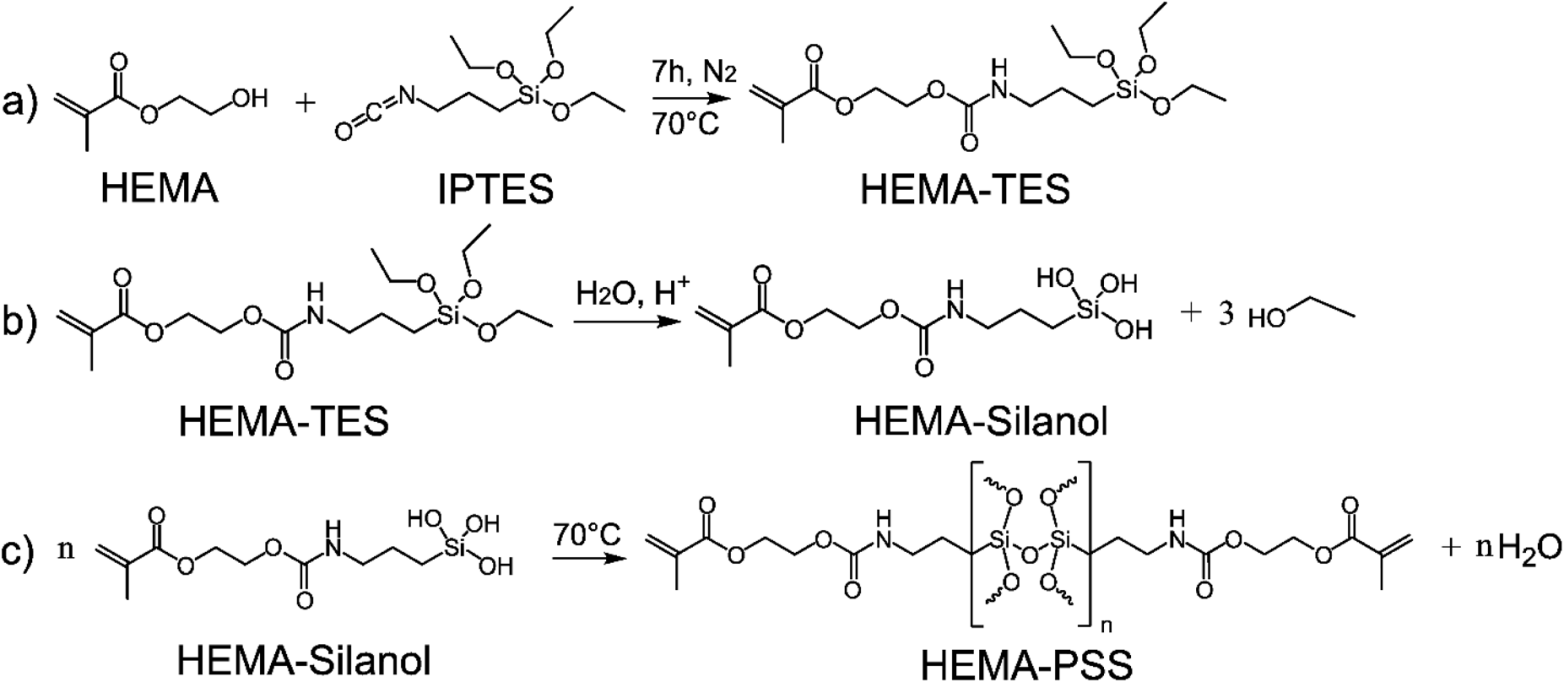
Schematic of HEMA-TES precursor synthesis and hydrolysis–condensation process. (a) Carbamate bond formation between 2 hydroxyethyl methacrylate (HEMA) and 3-isocyanatopropyltriethoxysilane (IPTES), producing the HEMA-TES precursor with both methacrylate and siloxane functional groups. (b) Acid-catalysed hydrolysis of triethoxysilane groups, yielding HEMA silanol and ethanol. (c) Subsequent condensation of silanol moieties leads to polysilsesquioxane (PSS) network formation in the hybrid material.

Four formulations were prepared with varying HEMA-TES content—0, 5, 10, and 20 wt% of PSS—and were designated as HT0, HT5, HT10, and HT20, respectively (Table 1). The weight of HEMA-TES used in each formulation was adjusted based on the stoichiometry of the hydrolysis–condensation reaction, which involves the release of three moles of ethanol per mole of HEMA-TES. Accordingly, a theoretical mass loss of 36.3% was considered for accurate composition calculation, as illustrated in Fig. 2b and c.

**Table 1:** Composition of the formulations with different siloxane content (PSS) for the preparation of PHEMA–PSS hybrids

Hybrid formulation	HEMA-TES (g)	HEMA (g)	HCl^a^ (g)	PI (g)
HT0 (0% PSS)	0	2.235	0.750	0.015
HT5 (5% PSS)	0.237	2.085	0.750	0.015
HT10 (10% PSS)	0.473	1.935	0.750	0.015
HT20 (20% PSS)	0.947	1.635	0.750	0.015

a 2 wt% HCl aqueous solution. PI: photoinitiator.

### 2.3. Hybrid formation

To prepare the hybrids, the formulations listed in Table 1 were poured into Teflon molds (30 mm in diameter, 4 mm in height) and exposed to LED light (365/405 nm, 40 W) for 4 min. Photopolymerization and photocrosslinking were considered complete when no flow was observed upon tilting the mold, indicating solidification of the material. During this process, methacrylate groups from both HEMA and HEMA–PSS underwent polymerization, resulting in the formation of poly(2-hydroxyethyl methacrylate)–polysilsesquioxane (PHEMA–PSS) hybrids.

Following polymerization, the hybrids were thoroughly washed with ethanol and then with ultrapure water to remove unreacted or uncrosslinked components. The cleaned hybrids were subsequently vacuum-dried and stored for further characterization and experimentation (Fig. S1).

### 2.4. *S*-Nitrosoglutathione (GSNO) synthesis

*S*-Nitrosoglutathione (GSNO) was synthesized following the method described by Vercelino *et al.*,^39^ with slight modifications. Briefly, 4.5 g of glutathione (gamma-glutamyl-cysteinyl-glycine; GSH) were dissolved in 23 mL of ultrapure water and 1 mL of concentrated HCl (37 wt%) in a beaker. The solution was cooled in an ice bath and protected from light. Subsequently, 1.0 g of sodium nitrite (NaNO_2_) was added under continuous magnetic stirring. Within approximately 1.5 min, the solution turned dark pink, indicating the formation of GSNO. To precipitate GSNO, 30 mL of cold acetone was added to the reaction mixture. The resulting precipitate was collected by vacuum filtration, washed with cold acetone to remove impurities, and lyophilized for 24 h. The dried GSNO powder was stored in a desiccator, protected from light, and kept in a freezer until use.

### 2.5. *S*-Nitroso-*N*-acetyl-dl-penicillamine (SNAP) synthesis

*S*-Nitroso-*N*-acetyl-dl-penicillamine (SNAP) was synthesized following the procedure described by Lautner *et al.*,^40^ with minor modifications. First, 2.0 g of sodium nitrite (NaNO_2_) were dissolved in 24 mL of ultrapure water to prepare an aqueous nitrite solution. In parallel, 2.0 g of *N*-acetyl-dl-penicillamine (NAP) were dissolved in a mixture of 40 mL methanol (MeOH), 2 mL concentrated sulfuric acid (H_2_SO_4_), and 8 mL concentrated hydrochloric acid (HCl) in a round-bottom flask. The NaNO_2_ solution was added dropwise to the NAP solution under continuous stirring. After approximately 10 min, the solution developed a dark green colour, indicating SNAP formation. The reaction mixture was then transferred to a 1 L beaker placed in an ice bath and protected from light.

To facilitate SNAP crystallization, a gentle stream of nitrogen gas was introduced into the beaker to promote gradual evaporation of methanol over approximately 12 h. The resulting SNAP crystals were collected by vacuum filtration, washed with cold ultrapure water, and vacuum-dried for 48 h. The final product was stored in a desiccator, protected from light, and kept in a freezer until use.

### 2.6. Incorporation of GSNO and SNAP into PHEMA–PSS hybrids

Dried PHEMA–PSS hybrids were immersed in 40 mmol L^−1^ of either aqueous GSNO solution or ethanolic SNAP solution for 24 h to allow swelling and passive impregnation of the NO donors. To minimize thermal degradation of the *S*-nitrosothiols during this process, the samples were kept refrigerated and protected from light throughout the incubation period. Following impregnation, GSNO- and SNAP-loaded hybrids were vacuum-dried for 48 h. All samples were subsequently stored in a desiccator, protected from light, and kept in a freezer until further analysis or use (Fig. S1).

### 2.7. Vibrational analysis

Fourier transform infrared spectroscopy with attenuated total reflectance (FTIR-ATR) was performed using an Agilent Cary 660 spectrometer. Spectra were acquired over the range of 4000 to 400 cm^−1^, with a resolution of 4 cm^−1^ and an average of 64 scans per sample to ensure adequate signal-to-noise ratio.

### 2.8. Proton nuclear magnetic resonance spectroscopy (^1^H NMR)

^1^H NMR spectra were recorded using a Bruker Avance 500 MHz spectrometer. Samples were prepared by dissolving approximately 15 mg of material in 700 µL of deuterated dimethyl sulfoxide (DMSO-d_6_). Chemical shifts are reported in parts per million (ppm) relative to the residual solvent peak.

### 2.9. Swelling degree (SD)

To evaluate the swelling behaviour, dried PHEMA–PSS hybrids were cut into discs weighing approximately 40 mg. The discs were immersed in either 1× phosphate-buffered saline (PBS) or ethanol and incubated in a water bath at either 25 °C or 37 °C. The swelling degree (SD) was calculated using eqn (1):

1 SD (%) = ((*W*_s_ − *W*_d_)/*W*_d_) × 100

where *W*_s_ is the weight of the swollen hybrid at a given time point, and *W*_d_ is the initial dry weight. Each measurement was performed in triplicate to ensure reproducibility.

### 2.10. Contact angle measurements

Surface wettability was assessed using the sessile drop method on an Attension optical tensiometer (Biolin Scientific). A droplet of ultrapure water (20 µL) was deposited onto the surface of the swollen hybrids samples to minimize additional water uptake during analysis, and the contact angle was measured after allowing the drop to stabilize for 120 seconds at 25 °C. Measurements were performed in triplicate to ensure accuracy and reproducibility.

### 2.11. Differential scanning calorimetry (DSC)

Differential scanning calorimetry (DSC) was performed using a Q2000 calorimeter (TA Instruments). Approximately 10 mg of each sample was sealed in standard aluminium pans. The samples were subjected to a heating–cooling cycle from 20 °C to 210 °C at a rate of 20 °C min^−1^, with 3-minute isothermal holds at both temperature extremes. All measurements were conducted under a nitrogen atmosphere to prevent oxidative degradation.

### 2.12. Determination of GSNO and SNAP loads and NO release profiles

Nitric oxide loading and release from the PHEMA–PSS hybrids were quantified using a chemiluminescence-based Nitric Oxide Analyzer (NOA 280i, Sievers, Boulder, CO, USA). The method is based on the gas-phase reaction between NO released from the sample and ozone (O_3_), which generates electronically excited nitrogen dioxide 
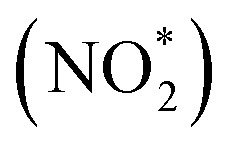
. Upon relaxation to the ground state, 
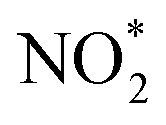
 emits chemiluminescent radiation, which is detected and quantified.

For total NO loading determination, approximately 40 mg (∼60 mm^2^) of dried hybrid discs were placed in the NOA reaction chamber containing 8 mL of 0.2 mol L^−1^ ascorbic acid and 2 mL of 1 mol L^−1^ NaOH (final pH ≈ 11.0). The samples were exposed to visible light at 37 °C to promote complete NO release from the incorporated RSNOs *via* the ascorbate reduction pathway. The instrument was calibrated using standard additions of a 10 mmol L^−1^ NaNO_2_ solution into a reducing solution of NaI (150 mg NaI in 10 mL water with 60 µL glacial acetic acid), as described by de Souza *et al.*^41^

For real-time NO release kinetics, dry hybrids were immersed in 10 mL of 1× phosphate-buffered saline (PBS, pH 7.4) with 1 mmol L^−1^ EDTA at 37 °C within the NOA reaction flask. Samples were kept in the dark to prevent photolysis, and NO release was monitored continuously under a nitrogen flow (7 torr). The cumulative NO release was calculated by integrating the area under the NO release curve. All measurements were performed in triplicate to ensure accuracy and reproducibility.

### 2.13. Endothelial cell culture

Human umbilical vein endothelial cells (HUVEC; Lonza #CC 2519) were maintained in EGBM 2 growth medium (Lonza #CC 3162) at 37 °C in a humidified atmosphere with 5% CO_2_. Upon reaching approximately 80% confluence, cells were washed with Hank's Balanced Salt Solution (Sigma-Aldrich #H6648) and detached using 0.25% trypsin EDTA (Gibco #25300054) for 2 min at 37 °C. Trypsin activity was halted by adding DMEM (Vitrocell #2325) supplemented with 10% fetal bovine serum (FBS; Vitrocell #S0011). Cells were counted using a Neubauer hemocytometer and reseeded into fresh culture vessels flasks with EGBM-2 medium for expansion or into culture plates to subsequent experiments.

HUVECs were seeded into culture plates and maintained in EGBM-2 medium until reaching confluence. Then, were exposed to PHEMA–5% PSS hybrid (HT5) discs loaded or not with SNAP (20 or 40 mM) for either 6 or 24 h. In another set of experiments, to induce an environment challenge,^42^ serum-starved HUVECs were exposed to HT5 discs loaded or not with SNAP (20 or 40 mM) for either 6 or 24 h. Control cells were maintained without hybrid discs in complete or serum-free medium. All treatments were performed at 37 °C in a 5% CO_2_ incubator.

After each treatment, wells were gently washed with 100 µL of phosphate-buffered saline (PBS). Then, cells were incubated with 100 µL of MTT solution (0.5 mg mL^−1^ in PBS) for 3 h at 37 °C in a 5% CO_2_ incubator. After incubation, the MTT solution was removed, and 100 µL of dimethyl sulfoxide (DMSO) was added to each well to dissolve the formazan crystals. Plates were gently shaken for 30 min, and the absorbance was measured at 570 nm using a SpectraMax M3 plate reader (Molecular Devices, USA). Cell viability was calculated as a percentage relative to untreated control cells.^43^

### 2.14. Details of theory/computation

Gas-phase molecular dynamics simulations were carried out at the GFN-FF level^44^ in *x*TB 6.6.1 software^45^ on NVT ensemble, at 298.15 K and a time step of 2 fs for a total simulation time of 1 ns. The simulated systems consisted of two 13-mer chains of HEMA forming a “sandwich” with GSNO or SNAP in the middle. Each chain length was constrained during the simulations at 25.9 Å in order to avoid chain folding. To increase conformational space sampling, two initial structures for each complex was built: (i) with all hydroxyl groups pointing towards the solute, named hereafter as “hydrophilic”, and a total separation of ∼20 Å between chains; and (ii) with all hydroxyl groups pointing outwards from the solute, named hereafter as “hydrophobic”, and a total separation of ∼13 Å between chains. The structure from the final frame of each simulation were refined through full optimization at the GFN2-*x*TB semi-empirical method.^46^ The binding Gibbs free energy (Δ*G*_bind_) from the optimized complexes were estimated using Autodock Vina 1.2.7 software^47,48^ with the improved Vinardo scoring function.^49^ In simulations with both GSNO and SNAP, the “hydrophilic” starting structures rendered more stable final complexes (with lower Δ*G*_bind_), and thus only those results are discussed in the main text. Trajectory analysis was made in VMD1.9.3 software,^50^ and images generated in Chimera1.18 (ref. 51).

### 2.15. Data analysis

Quantitative data are reported as mean ± standard error of the mean (SEM). Statistical analysis was conducted using OriginPro 8.5 and GraphPad Prism version 8.0. Comparisons between two groups were performed using Student's *t*-test, while comparisons across three or more groups employed one-way analysis of variance (ANOVA), followed by appropriate post hoc tests. Statistical significance was defined as *p* < 0.05.

## 3. Results and discussion

### 3.1. Synthesis of the HEMA-TES precursor

The synthesis of the hybrid precursor HEMA-TES was achieved through the reaction of 2-hydroxyethyl methacrylate (HEMA) with 3-isocyanatopropyltriethoxysilane (IPTES), yielding a molecule with both methacrylate and triethoxysilane functional groups, as illustrated in Fig. 2. The reaction involves the formation of a carbamate linkage between the hydroxyl group of HEMA and the isocyanate group of IPTES (Fig. 2a). Subsequent hydrolysis of the triethoxysilane moiety generates HEMA-silanol and ethanol (Fig. 2b), which can then condense to form polysilsesquioxane (PSS) networks within the hybrid material (Fig. 2c).

**Fig. 3:**
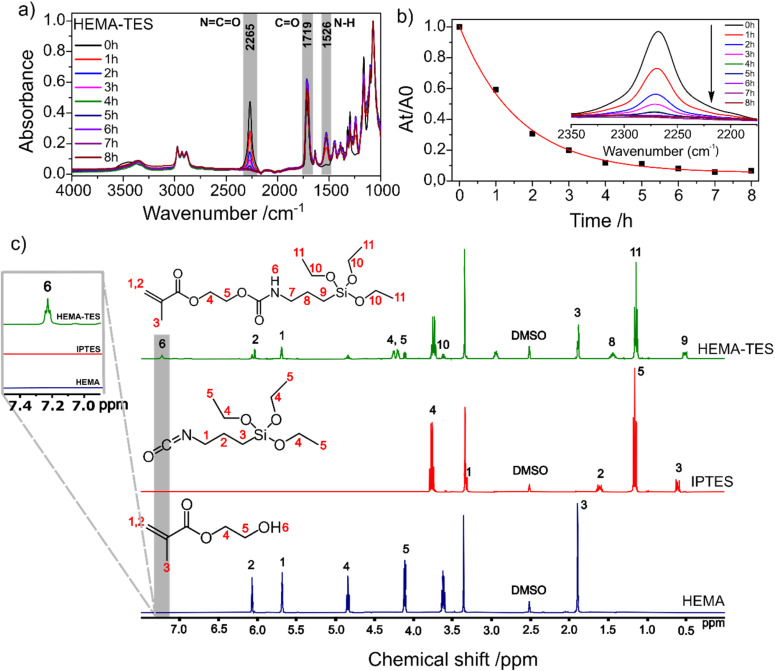
Spectroscopic confirmation of HEMA-TES formation. (a) FTIR ATR spectra showing gradual disappearance of the isocyanate (N=C=O) stretching band at 2265 cm^−1^, along with the emergence of characteristic carbamate bands at 1526 cm^−1^ (N–H stretch) and 1719 cm^−1^ (C=O stretch), during the reaction. (b) Kinetic plot (*A*_*t*_/*A*_0_*vs.* time) for NCO consumption, demonstrating reaction completion in ∼7 h. (c) ^1^H NMR spectra of HEMA-TES (top), IPTES (middle), and HEMA (bottom), highlighting the new carbamate proton peak at 7.3 ppm in HEMA-TES, confirming conjugation.

The progression of the reaction was monitored by FTIR-ATR spectroscopy, focusing on the isocyanate stretching band at 2265 cm^−1^.^52^ As shown in Fig. 3a, the gradual decrease in the intensity of this band indicates the consumption of the NCO functional group. Concurrently, new absorption bands emerged, including an N–H stretching band at 1526 cm^−1^ and an increased intensity of the carbonyl band at 1719 cm^−1^, both characteristic of carbamate formation. The reaction kinetics were further analysed by plotting the normalized decrease in absorbance of the NCO band over time (*A*_*t*_/*A*_0_), revealing that the reaction reached completion in approximately 7 h (Fig. 3b).

Complementary structural confirmation was obtained *via*^1^H NMR spectroscopy (Fig. 3c). The spectrum of IPTES displayed characteristic peaks at *δ* 0.50 ppm (Si–CH_2_, *δ*3), 1.18 ppm (CH_3_, *δ*5), 1.55 ppm (CH_2_, *δ*2), 3.31 ppm (NCO-adjacent CH_2_, *δ*1), and 3.85 ppm (alkoxy CH_2_, *δ*4)^52,53^. The HEMA spectrum showed signals at 1.92 ppm (CH_3_, *δ*3), 4.15 ppm (OH-adjacent CH_2_, *δ*5), 4.85 ppm (ester CH_2_, *δ*4), and vinyl protons at 5.70 ppm (*δ*1) and 6.02 ppm (*δ*2)^54,55^. The HEMA-TES spectrum revealed a distinct new peak at 7.3 ppm (*δ*6), corresponding to the NH proton of the newly formed carbamate group (NH(CO)O), which is absent in the spectra of the individual reactants. This finding confirms successful conjugation and the formation of the HEMA-TES hybrid precursor.

### 3.2. Preparation of PHEMA–PSS hybrids

**Fig. 4:**
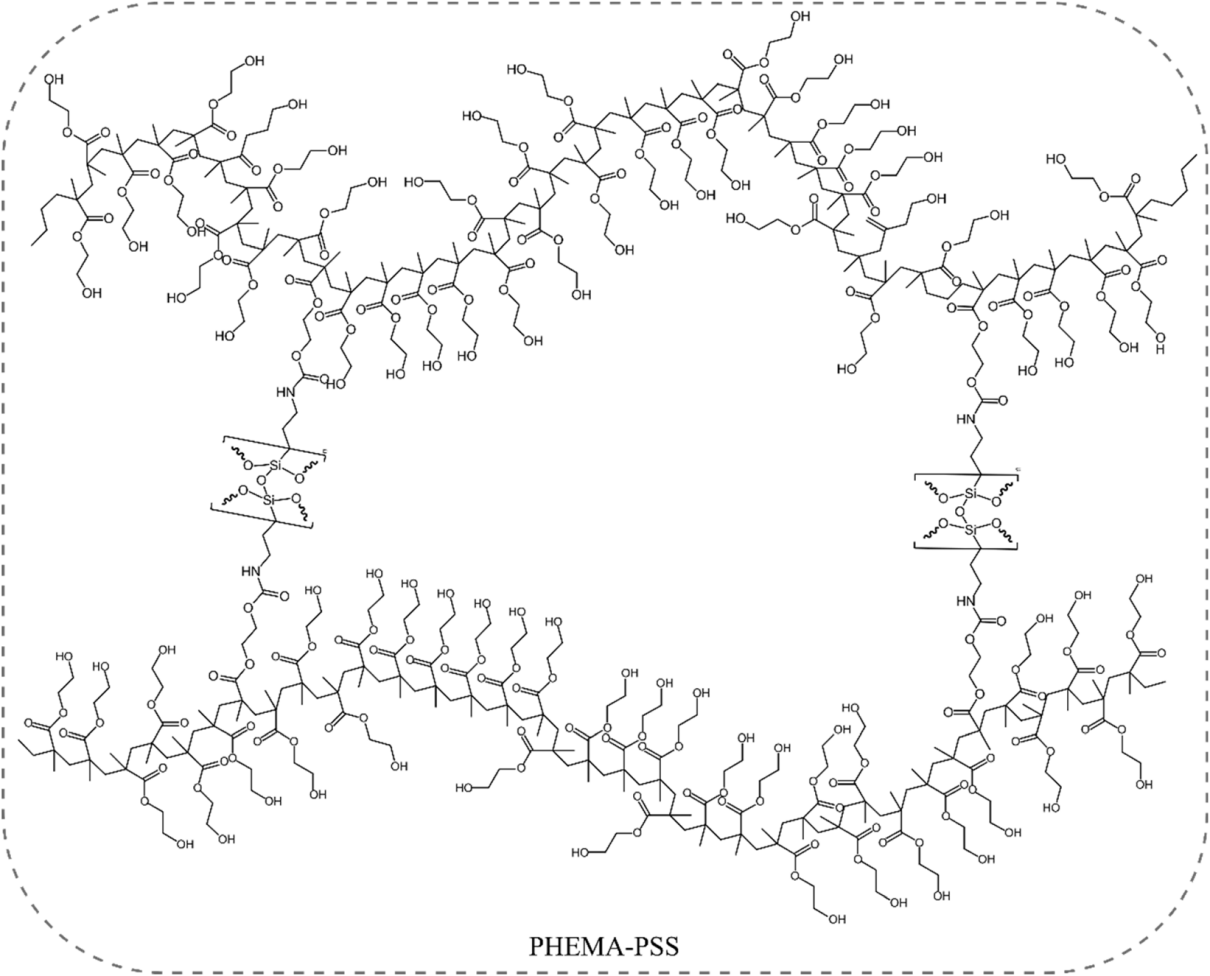
Schematic representation of the structure of the photo-crosslinked PHEMA–PSS network.

To investigate the structural influence of PSS content, hybrid formulations were prepared with varying concentrations of the siloxane-containing precursor HEMA-TES, as summarized in Table 1. In each formulation, PSS was synthesized *in situ* through the hydrolysis and condensation of HEMA-TES (see Fig. 2b and c) and then copolymerized with free HEMA *via* photoinitiated crosslinking to form PHEMA–PSS hybrids (Fig. 4).

The control formulation, HT0, which lacked HEMA-TES and thus did not have PSS, displayed poor photocrosslinking after 4 min of light exposure. The resulting material remained soft, with a sticky and uneven surface, likely due to the absence of any crosslinking agent, leading to linear PHEMA chains with low mechanical integrity. In contrast, formulations HT5, HT10, and HT20, which contained increasing amounts of HEMA-TES, yielded hybrids with greater rigidity and smoother surfaces, facilitating removal from the mold. These improvements are attributed to the formation of the PSS network acting as an inorganic crosslinker, enhancing the mechanical strength and structural uniformity of the hybrid materials.

**Fig. 5:**
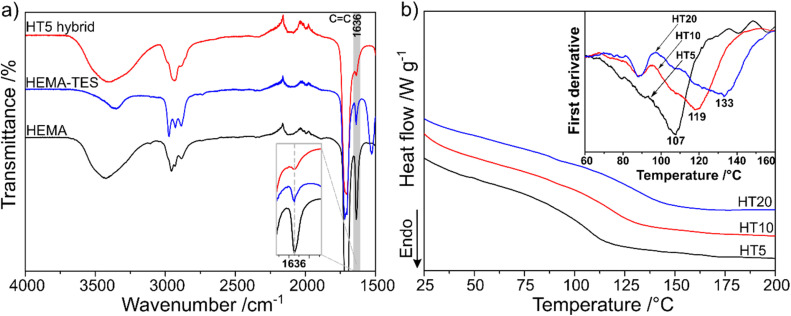
Spectroscopic and thermal characterization of PHEMA–PSS hybrids. (a) FTIR-ATR spectra of HEMA (black), HEMA-TES (blue), and HT5 hybrid (red). In the HT5 spectrum, the vinyl C=C stretching band at 1636 cm^−1^ is nearly absent, confirming effective photopolymerization and crosslinking. (b) Differential scanning calorimetry (DSC) thermograms of HT5 (black), HT10 (red), and HT20 (blue) hybrids. Glass transition temperatures (*T*_g_), determined from thermogram inflection points or the minima in the first-derivative curves, systematically increase from 107 °C (HT5) to 119 °C (HT10) and 133 °C (HT20), reflecting enhanced network rigidity due to increasing polysilsesquioxane crosslinker content.

Fig. 5a shows the FTIR-ATR spectra of pure HEMA, HEMA-TES, and the HT5 hybrid. A notable reduction in the intensity of the vinyl C=C stretching band at 1636 cm^−1^ is observed in the HT5 spectrum, indicating effective consumption of the double bonds during photopolymerization and confirming successful crosslinking and network formation in the hybrid system.

Fig. 5b shows the DSC thermograms obtained in the second heating cycle and the corresponding first derivative curves for hybrids with different siloxane contents to evaluate their influence on the thermal properties. The thermograms revealed a clear upward shift in glass transition temperatures (*T*_g_) with increasing siloxane content. *T*_g_ was determined from the inflection point of the thermogram or the minimum of its first derivative, yielding values of 107 °C, 119 °C, and 133 °C for HT5, HT10, and HT20, respectively. This trend aligns with expectations: as the proportion of rigid inorganic PSS crosslinkers increases, the resulting network becomes less elastomeric, requiring higher temperatures to achieve polymer chain mobility.

### 3.3. Swelling degree and wettability of PHEMA–PSS hybrids

To evaluate the solvent absorption capacity of the PHEMA–PSS hybrids for NO donor loading, the swelling behaviour of HT5, HT10, and HT20 formulations was characterized in both phosphate-buffered saline (PBS) and ethanol at 25 °C over 24 h. The swelling degree reflects the network's ability to accommodate solvent molecules and is thus indicative of pore accessibility and crosslinking density.

**Fig. 6:**
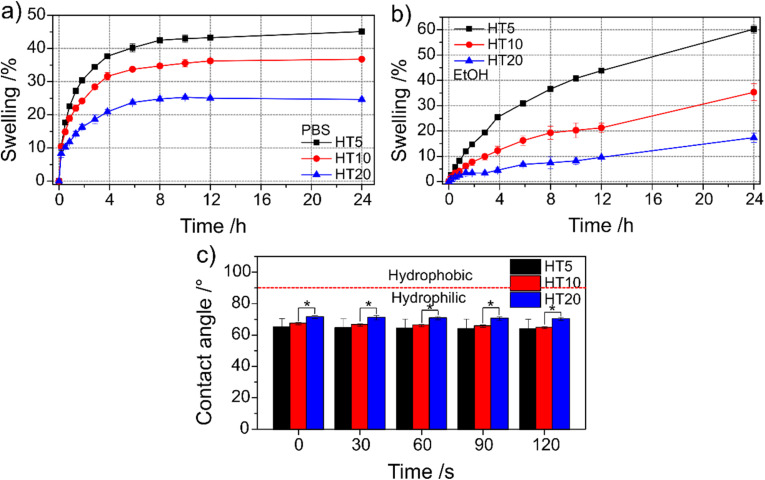
Swelling and surface wettability of PHEMA–PSS hybrids. (a) Swelling kinetics of HT5, HT10, and HT20 hybrids in PBS at 37 °C, showing equilibrium swelling values reached within 12 h. (b) Swelling kinetics in ethanol at 25 °C, where swelling progressed over the full 24-hours period without reaching equilibrium. (c) Contact angle measurements over time (0–120 s) with ultrapure water droplets on HT5, HT10, and HT20 hybrid surfaces (*p < 0,0).

Fig. 6a and b show the swelling kinetics in PBS and ethanol, respectively. In PBS, all three hybrid compositions reached equilibrium swelling within approximately 12 h, with final swelling degrees of 45.1 ± 0.6% (HT5), 36.8 ± 0.2% (HT10), and 24.6 ± 0.6% (HT20). These values are consistent with the typical swelling behaviour reported for PHEMA-based materials, which range between 10% and 60%.^56–58^ In contrast, swelling in ethanol continued to progress beyond 24 h, without clearly reaching equilibrium. After 24 h, the ethanol-swollen hybrids exhibited swelling degrees of 60.2 ± 1.5% (HT5), 35.3 ± 3.3% (HT10), and 17.4 ± 0.6% (HT20).

Notably, while HT10 displayed comparable swelling in both PBS and ethanol (36.8% and 35.3%, respectively), HT20 absorbed less ethanol than water, and HT5 absorbed more ethanol than water. The increased ethanol uptake in HT5 is consistent with prior studies showing that PHEMA hydrogels exhibit greater swelling in ethanol compared to water,^59^ and that PHEMA homopolymers display higher solubility in polar organic solvents.^60,61^ However, in this hybrid system, the presence of a PSS network significantly influences swelling behaviour. As the PSS content increases, so does the crosslinking density, which restricts chain mobility and solvent uptake. This trend is evident in both solvents, with swelling degrees decreasing consistently from HT5 to HT20.

Fig. 6c shows water contact angle measurements over 120 seconds on the surface of each hybrid composition. Across all time points (0–120 s), the contact angles remained between 60° and 70°, indicating that all materials are hydrophilic (*θ* < 90°).^62^ Statistically significant differences were observed only between HT10, and HT20 (*p* < 0,05).

The hydrophilic character of these materials is a desirable trait for biomedical applications, particularly for blood-contacting devices. Hydrophilic surfaces are known to promote improved tissue integration and reduce foreign body responses. Moreover, they are essential for minimizing protein adsorption and platelet adhesion, both of which are key steps in thrombus formation.^63^

### 3.4. GSNO and SNAP loadings and NO release profiles of PHEMA–PSS hybrids

**Fig. 7:**
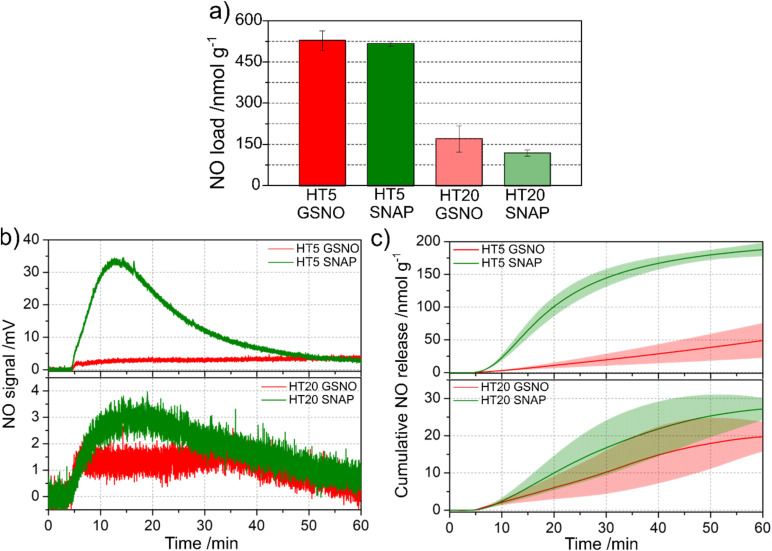
(a) Total NO loading, (b) real-time NO release, and (c) cumulative NO release of hybrids HT5 and HT20 loaded with 40 mmol L^−1^ of GSNO and SNAP solutions.

To characterize the NO release profiles from GSNO and SNAP incorporated into PHEMA–PSS hybrids, we selected the hybrid compositions with the lowest and highest PSS: HT5, and HT20 content, respectively. Fig. 7a shows the GSNO and SNAP loads obtained in the impregnation of the HT5 and HT20 with aqueous GSNO solution and ethanolic SNAP solution over 24 h. The hybrids with lower siloxane content (HT5) exhibited statistically equal (*p* > 0.05) GSNO and SNAP loads of ca. 520 nmol g^−1^, which are ca. 3 to 4 times higher than the loads obtained in the hybrids with higher siloxane content (HT20) for which the GSNO and SNAP charges obtained are also statistically equal (*p* > 0.05) with an average value of 143 nmol g^−1^. This result is in accordance with the observed higher swelling degrees of the HT5 hybrid, compared to the HT20 hybrid (Fig. 6a and b). The increase in the swelling degree of HT5 compared to HT20 is directly correlated with the higher GSNO and SNAP loading of HT5, relative to HT20. Fig. 7b shows the real-time NO release curves of GSNO and SNAP from HT5 (top curve) and HT20 (bottom curve) hybrids.

The first aspect that draws attention is that in the HT5/SNAP hybrid, displays a much higher and increasing NO flux than the HT5/GSNO hybrid, up to 15 min after hydration. From this time ahead, the NO flux of the HT5/SNAP hybrid starts decaying, reaching a flux similar to that of HT5/GSNO hybrid, after 45 min. In contrast, the NO flux of the HT5/GSNO remains low and nearly constant from the beginning up to the end of the measurement at 60 min. Similar profiles are obtained for the real time NO release curves of SNAP and GSNO incorporated in the HT20 hybrid, although at much lower NO fluxes, in accordance with the lower SNAP and GSNO charges in this hybrid. In this case, the NO flux also increases from the beginning up to *ca.* 10 min and then starts to decrease reaching the NO flux displayed by the HT20/GSNO hybrid, after *ca.* 35 min, which is lower since the beginning. Therefore, the higher NO release rates from both GSNO and SNAP in HT5 compared to HT20 are directly correlated with the higher swelling degree of HT5 relative to HT20, which leads to greater GSNO and SNAP loading and, consequently, to higher NO release rates for HT5 than for HT20, as expected.

The lower NO fluxes displayed by the HT5 and HT20 hybrids charged with GSNO, relative to those displayed by the HT5 and HT20 hybrids charged with SNAP is in accordance with the results reported by Melvin *et al.*^64^ and Fan *et al.*^65^ that showed that aqueous GSNO exhibits greater stability than aqueous SNAP solutions under common laboratory conditions at various pH, temperature, and light exposure conditions.

These profiles are reflected in the corresponding cumulative NO release curves of Fig. 7c. These curves show that in the first 20 min the rate of NO release of the HT5/SNAP hybrid is *ca.* 10 times higher than that of the HT5/GSNO hybrid (7.98 ± 1.07 nmol g^−1^ min^−1^ × 0.79 ± 0.36 nmol g^−1^ min^−1^, corresponding to 578 pmol cm^−2^ min^−1^ and 52 pmol cm^−2^ min^−1^, respectively). After 15 min, the rate of NO release of the HT5/SNAP hybrid starts decreasing and becomes equal to the rate of NO release of the HT5/GSNO hybrid, which is constant since the beginning (0.79 nmol g^−1^ min^−1^).

The corresponding cumulative NO release curves of the HT20/SNAP and HT20/GSNO displays much lower, and nearly constant rates of NO release over the 55 min time of monitoring (0.77 ± 0.38 nmol g^−1^ min^−1^ and 0.39 ± 0.24 nmol g^−1^ min^−1^, corresponding to 54 pmol cm^−2^ min^−1^ and 23 pmol cm^−2^ min^−1^, respectively). Replicates of the real time and cumulative NO release profiles are shown in Fig. S2 and S3.

The NO release rate needed for antithrombotic activity is reported to be in the range of 18–36 fmol min^−1^ cm^−2^.^24,66^ Therefore, both HT5 and HT20 hybrids charged with 520 nmol g^−1^ and 143 nmol g^−1^, respectively, exhibit rates of NO release well above the antithrombotic range and have the potential to be used as antithrombogenic blood contacting materials.

### 3.5. Computational modelling of GSNO and SNAP intermolecular interactions with PHEMA–PSS

The swelling profiles of the PHEMA–PSS hybrids in both aqueous (PBS) and ethanol media enabled the absorption of hydrophilic GSNO and hydrophobic SNAP, respectively. Theoretical modelling was conducted to better understand the molecular interactions between these NO donors and the polymeric matrix.

Glutathione (GSH), from which GSNO is derived, is known to form intramolecular and intermolecular hydrogen bonds *via* its protonated carboxylic acid groups and zwitterionic salt bridges.^67^ These interactions govern its conformational stability, self-assembly behaviour, and biological function, including its role as an intracellular antioxidant.^68^ Upon *S*-nitrosation, forming GSNO, the –SNO moiety is not expected to interfere significantly with these hydrogen bonding interactions. When GSNO is synthesized from GSH using nitrous acid at pH ∼2, the molecule exists predominantly in a zwitterionic state, characterized by partial protonation of the carboxyl groups and full protonation of the amino group. During impregnation of the PHEMA–PSS hybrid, the GSNO solution is typically at pH ∼3, a condition in which GSNO maintains multiple hydrogen bonding donor (–NH_3_^+^, –NH amide, –OH) and acceptor (–COO^−^, C=O, –SNO) sites. These interactions promote strong solvation in water and enable its homogeneous absorption into hydrophilic environments of the PHEMA matrix.^69^

**Fig. 8:**
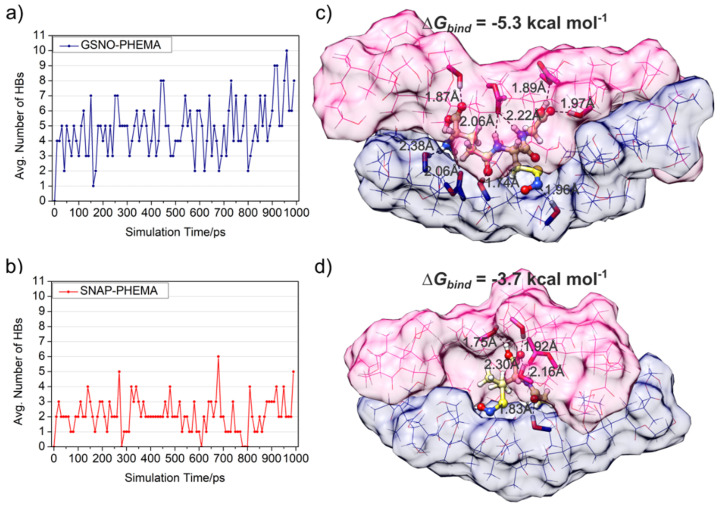
Average number of hydrogen bonds between PHEMA chain and (a) neutral GSNO and (b) neutral SNAP during 1 ns molecular dynamics simulation at the GFN-FF theoretical level. (c) Final structures from MDs of neutral GSNO–PHEMA/PSS complex and (d) neutral SNAP—PHEMA/PSS complex. Distances are reported in angstroms.

After absorption of the aqueous GSNO solution by the PHEMA–PSS hybrid, followed by water removal by vacuum drying, GSNO becomes immobilized into the PHEMA–PSS matrix through hydrogen bonding interactions with the pendant hydroxyl groups of the hydroxyethyl side chains of PHEMA. Each GSNO molecule has 6 HB donor sites (among N–H and O–H bonds) and 7 HB acceptor sites (among carbonyls and the NO and OH groups), allowing for the formation of multiple hydrogen-bonded interactions with the matrix, resulting in a solid solution of GSNO within the PHEMA–PSS hydrophilic microdomains. A similar mechanism was reported for GSNO incorporation into polyacrylic acid/Pluronic F127 hydrogels crosslinked by bisacrylamide.^70^ The formation of such a solid solution is supported by molecular dynamics simulations (Fig. 8a, S4 and S5), which show GSNO stabilized by up to 10 hydrogen bonds within a well-defined hydrophilic environment between two PHEMA chains of the matrix and a binding Gibbs free energy (Δ*G*_bind_) of −5.3 kcal mol^−1^ (Fig. 8c).

Upon rehydration, these hydrogen bonds are disrupted, and both the GSNO and the hydroxyl groups of PHEMA become re-solvated by water molecules. This hydration process confers diffusional mobility to GSNO molecules, which can then undergo encounters that lead to their bimolecular dimerization reaction with the formation of oxidized glutathione, GS-SG and the release of free NO. The same process led to the dimerization of SNAP with the release of free NO.

In contrast, SNAP, which is also synthesized *via S*-nitrosation with nitrous acid, is obtained in its neutral (acid) form, featuring a protonated carboxyl group. When SNAP is loaded into the PHEMA–PSS matrix from ethanol, its lower hydrophilicity compared to GSNO leads to fewer hydrogen bond interactions with PHEMA chains, whose hydroxyl groups form internal hydrogen bonds or interact preferentially with ethanol, while the methyl groups align toward the SNAP molecules, facilitating hydrophobic interactions (Fig. 8b, S6 and S7). This condition is also supported by gas-phase MD simulations that shows that fewer hydrophilic contacts ultimately lead to a weaker binding (Δ*G*_bind_ = −3.7 kcal mol^−1^, Fig. 8d).

Therefore, considering the various hydrogen bonds established between GSNO and the PHEMA chains, with stronger binding, relative to the mainly hydrophobic and weaker interactions between SNAP and the PHEMA chains, one may expect that GSNO faces more important diffusional restrictions to undergo dimerization during the hydration process than SNAP, what is in accordance with the lower NO release rate displayed by GSNO, compared to SNAP.

This dual behaviour, homogeneous release from GSNO and burst-release from SNAP, highlights the impact of microdomain distribution on NO delivery kinetics. The different rates of NO release obtained with GSNO and SNAP incorporated into PHEMA–PSS hybrids, as well as the use of hybrids with different PSS contents (*e.g.* HT5 and HT20), provides novel strategies for tuning the rate NO delivered locally to tissues, for stimulating cell proliferation or exerting microbicidal action or released from the surface of blood contacting antithrombogenic medical devices made of PHEMA–PSS.

### 3.6. Biocompatibility evaluation

**Fig. 9:**
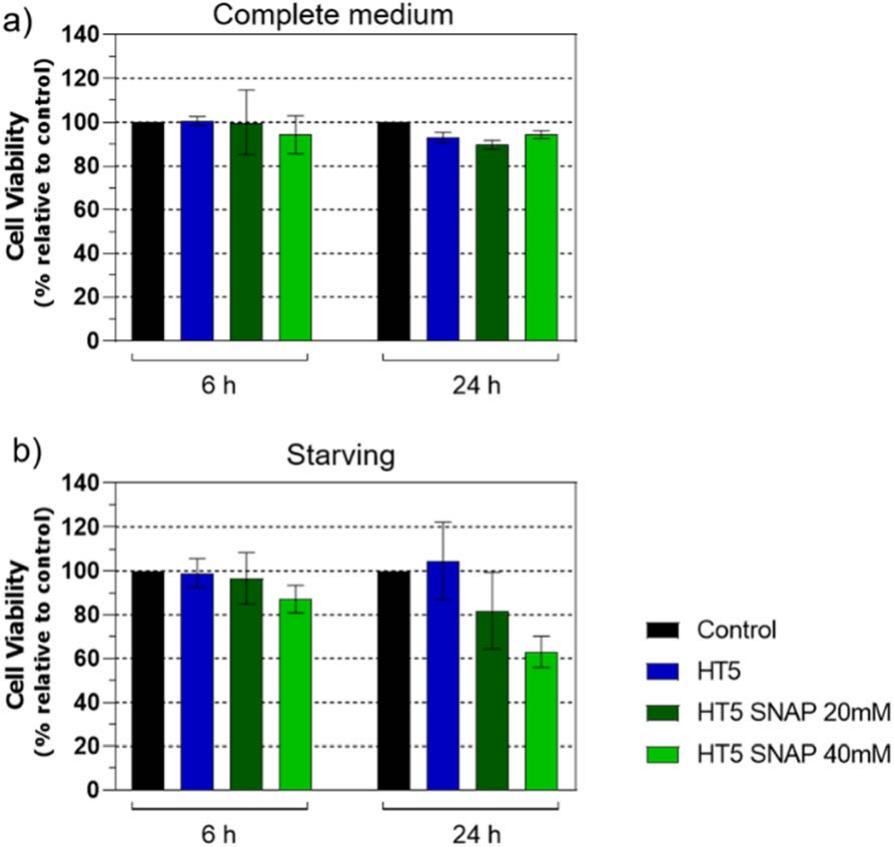
(a) Viability of HUVECs under control conditions or after incubation with HT5 discs, with or without SNAP (20 mM or 40 mM), for 6 h or 24 h. (b) Cell viability under the same conditions after 24 h of nutrient deprivation (starvation) as a stressor. Data are expressed as percentage relative to control. *N* = 3. Values are presented as mean ± SEM. One-way ANOVA: *P* > 0.05.

Biocompatibility assessment was conducted using the HT5/SNAP hybrid, the composition exhibiting the most efficient NO release (Fig. 7b). Two SNAP loadings (20 mM and 40 mM) were evaluated. Endothelial cell viability, assessed after 6 and 24 h exposure, remained unaffected either by HT5 alone or SNAP-load HT5 at both concentrations, indicating acceptable initial cytocompatibility (Fig. 9a and S8). Even under starving, HT5 did not compromise cell viability at 6 or 24 h (Fig. 9b and S8). While SNAP presence similarly showed no statistically significant toxicity, a trend toward reduced viability emerged at 24 h with 40 mM SNAP (Fig. 9b).

This may reflect NO accumulation-induced cytotoxicity, especially as the culture medium was not replenished during the assay. Indeed, prolonged NO exposure can impair mitochondrial respiratory complexes in endothelial cells, without immediate cell death, but potentially compromising function.^71^ Moreover, NO-mediated cytotoxicity displays threshold effects, where both steady-state concentration and cumulative exposure critically determine outcomes such as apoptosis or genotoxicity.^72^ Importantly, *in vivo* conditions, NO released from SNAP within a blood-contacting material would be rapidly cleared by flowing blood, preventing the localized NO accumulation observed *in vitro*, highlighting a limitation of our current protocol. Overall, HT5 without SNAP did not significantly reduce endothelial viability across all tested conditions (Fig. 9), reinforcing its potential as a biocompatible scaffold. Future studies might explore dynamic flow conditions and real-time NO clearance to better simulate physiological environments.

## 4. Conclusions

In this study, we developed and characterized a series of photocrosslinked hybrids composed of poly(2-hydroxyethyl methacrylate) (PHEMA) and polysilsesquioxane (PSS) networks for the controlled delivery of nitric oxide (NO). By tuning the content of the siloxane PSS, we modulated the physical properties of the materials, including thermal stability, and solvent-swelling behaviour. All formulations maintained hydrophilic surfaces, a key requirement for hemocompatible biomaterials. These networks allowed for the successful incorporation of two distinct NO donors, hydrophilic *S*-nitrosoglutathione (GSNO) and hydrophobic *S*-nitroso-*N*-acetyl-dl-penicillamine (SNAP), *via* passive absorption from aqueous and ethanolic media, respectively. Molecular modelling revealed that GSNO preferentially interacts with the PHEMA matrix through multiple hydrogen bonds, resulting in a hydration-dependent NO release profile. In contrast, SNAP exhibited weaker interactions with the PHEMA chains, leading to an accelerated early stage of NO release. This ability to modulate NO release kinetics by manipulating NO donor type and polymer microstructure offers a versatile platform for tailoring therapeutic NO delivery. The hybrids' favourable swelling behaviour, surface hydrophilicity, and cytocompatibility with endothelial cells further support their potential application as antithrombogenic coatings for blood-contacting medical devices. Together, these findings provide a foundation for the rational design of multifunctional biomaterials capable of delivering NO.

## Author contributions

H. V. A.: investigation, methodology, data curation, validation, formal analysis, writing – original draft; L. C. E. S.: investigation, methodology, formal analysis; B. R. G. S.: methodology, formal analysis; B. A. P.: methodology, formal analysis; D. M. G.: formal analysis and manuscript writing; A. P. C. D.: writing – review and editing; R. A. C.: writing – review and editing; M. G. O.: funding acquisition, conceptualization, supervision, writing – review and editing.

## Conflicts of interest

There are no conflicts to declare.

## Supplementary Material

RA-015-D5RA07870A-s001

## Data Availability

All the data supporting the findings of this study are available within the paper and its supplementary information (SI). Supplementary Information is available. See DOI: https://doi.org/10.1039/d5ra07870a.
