# Supplementary material for: Nitric oxide-releasing PHEMA/polysilsesquioxane photocrosslinked hybrids
Source: RSC Adv. 2025 Nov 18;15(53):45048–60. doi: 10.1039/d5ra07870a (PMC12624852; doi:10.1039/d5ra07870a)
Supplement: RA-015-D5RA07870A-s001 [file RA-015-D5RA07870A-s001.pdf]

Supplementary Information (SI) for RSC Advances.
This journal is © The Royal Society of Chemistry 2025

Nitric Oxide-Releasing PHEMA/Polysilsesquioxane Photocrosslinked Hybrids

Herllan Vieira de Almeida[a], Laura Caetano Escobar da Silva[a], Bruno de Almeida Piscelli[a], Beatriz Rafaelle Goes dos Santos[b], Daniele Mendes Guizoni[b], Ana Paula Couto Davel[b*], Rodrigo Antonio Cormanich[a], Marcelo Ganzarolli de Oliveira[a*]

[a]Institute of Chemistry, University of Campinas, UNICAMP, Campinas, 13083-970, SP, Brazil

[b]Institute of Biology, University of Campinas, UNICAMP, Campinas, 13083-862, SP, Brazil

Supplementary Information

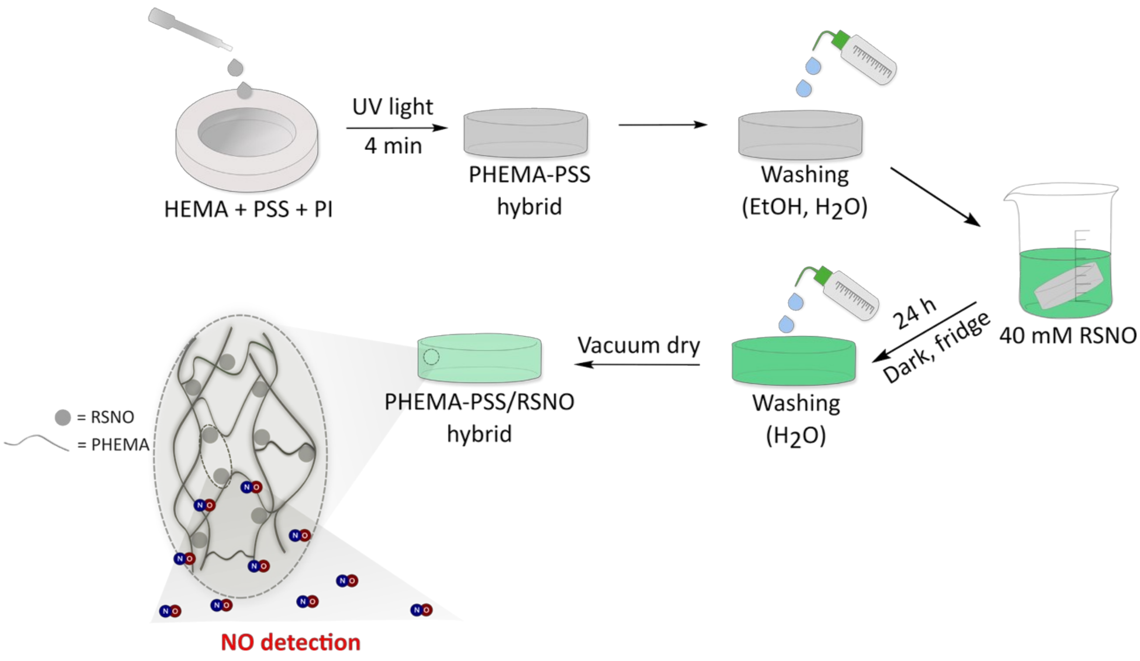

Fig. S1 Schematic diagram showing the steps involved in the PHEMA-PSS hybrid formation, RSNO (GSNO and SNAP) incorporation and nitric oxide (NO) detection.

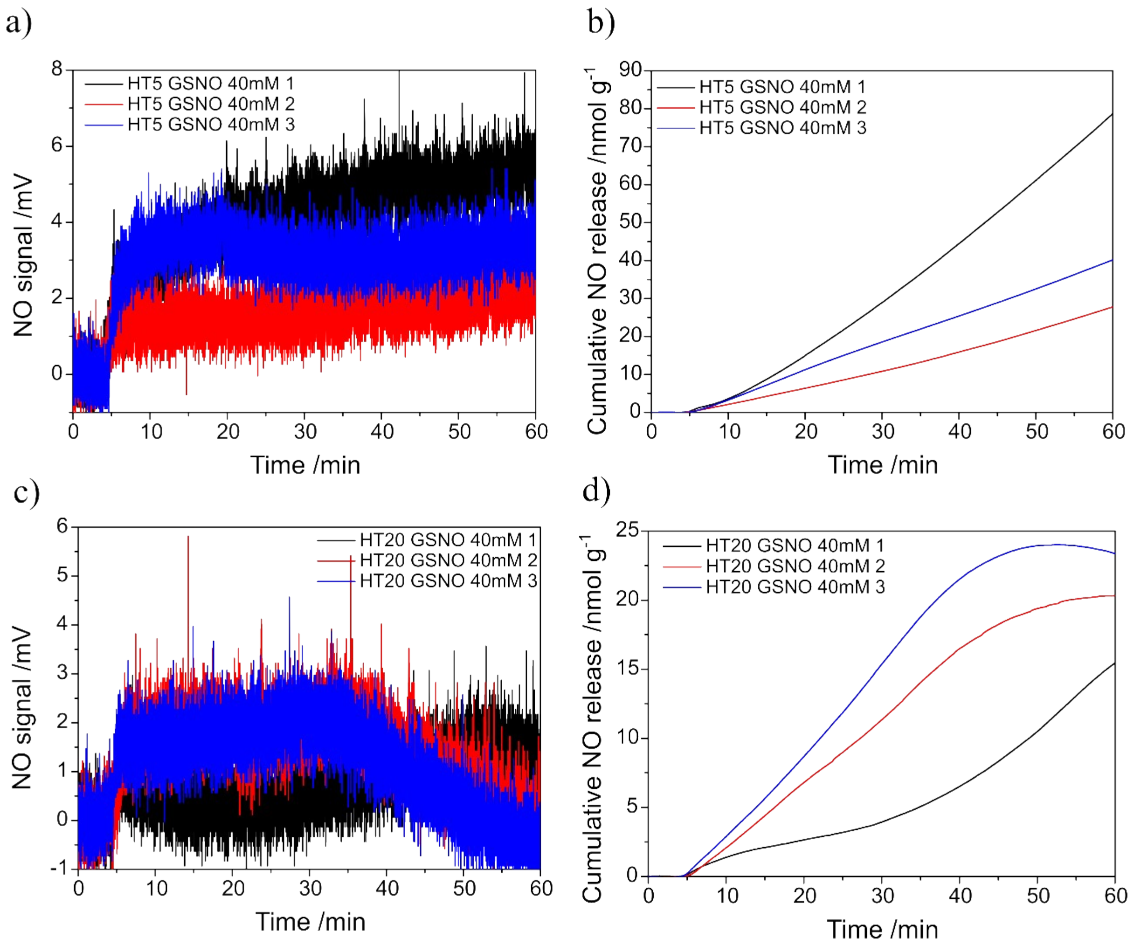

Fig. S2 (a) Real time NO release, and (b) Cumulative NO release of HT5 hybrid hydrogels loaded with 40 mmol L^{-1} of GSNO solution. (c) Real time NO release, and (d) Cumulative NO release of HT20 hybrid hydrogels loaded with 40 mmol L^{-1} of GSNO solution.

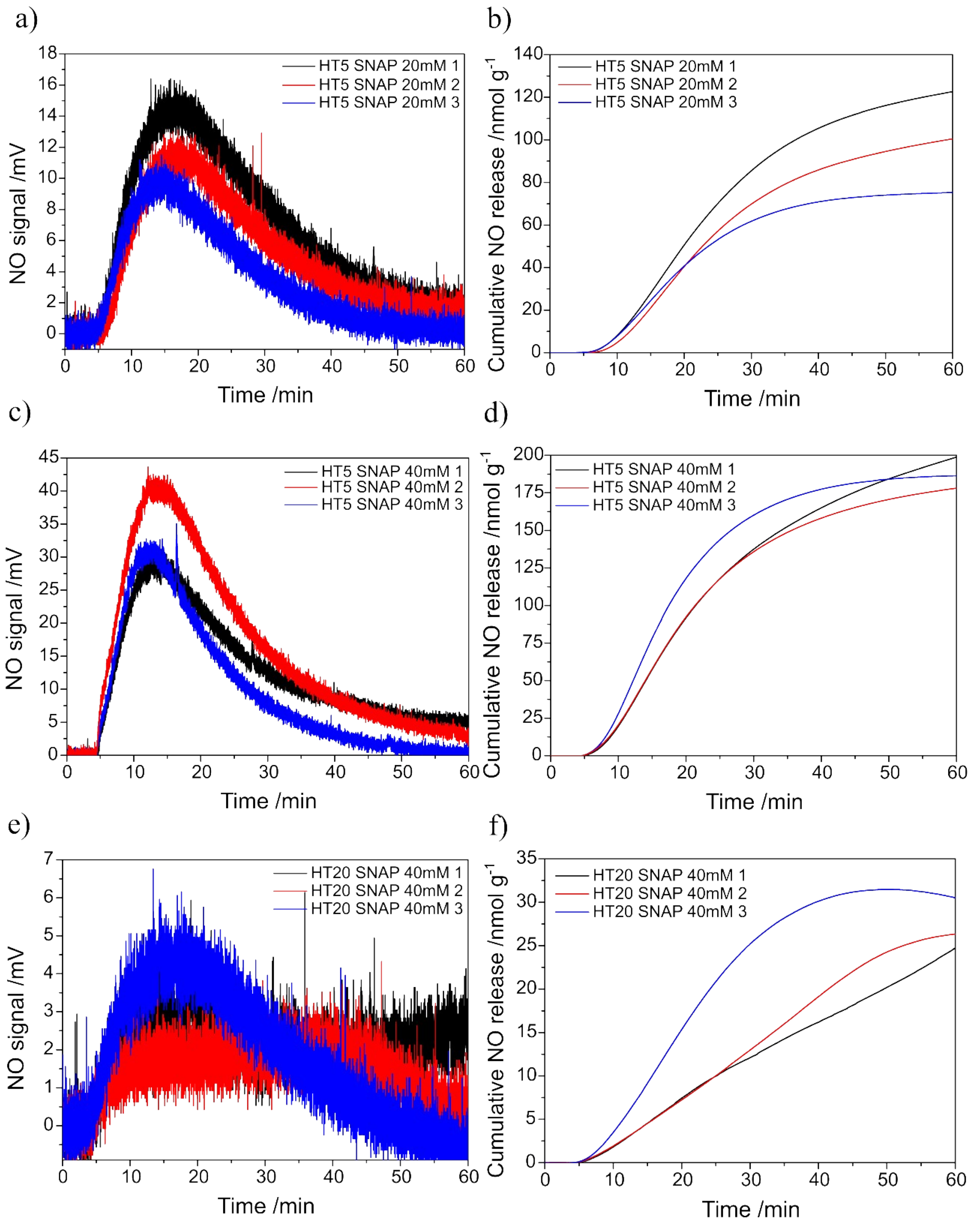

Fig. S3 (a) Real time NO release, and (b) Cumulative NO release of HT5 hybrid hydrogels loaded with 20 mmol L^{-1} of SNAP ethanolic solution. (c) Real time NO release, and (d) Cumulative NO release of HT5 hybrid hydrogels loaded with 40 mmol L^{-1} of SNAP ethanolic solution. (e) Real time NO release, and (f) Cumulative NO release of HT20 hybrid hydrogels loaded with 40 mmol L^{-1} of SNAP ethanolic solution.

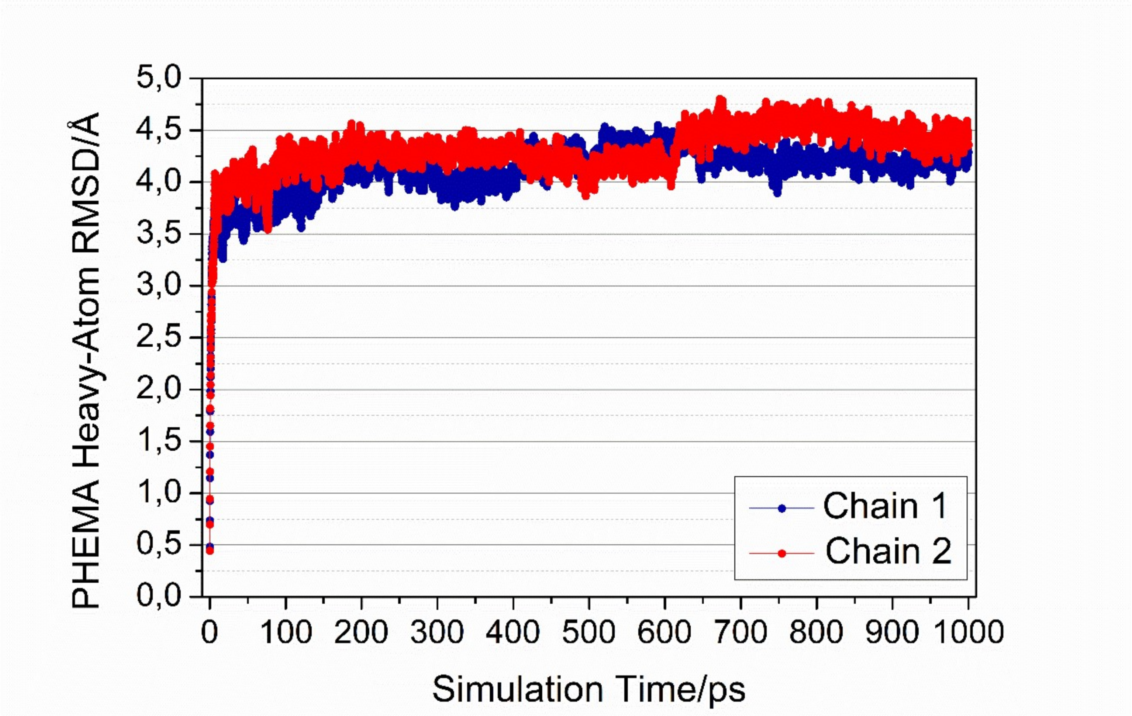

Fig. S4 Root mean square deviation (RMSD, in Å) over heavy-atoms from PHEMA chains for the "hydrophilic" PHEMA-GSNO 1 ns simulation.

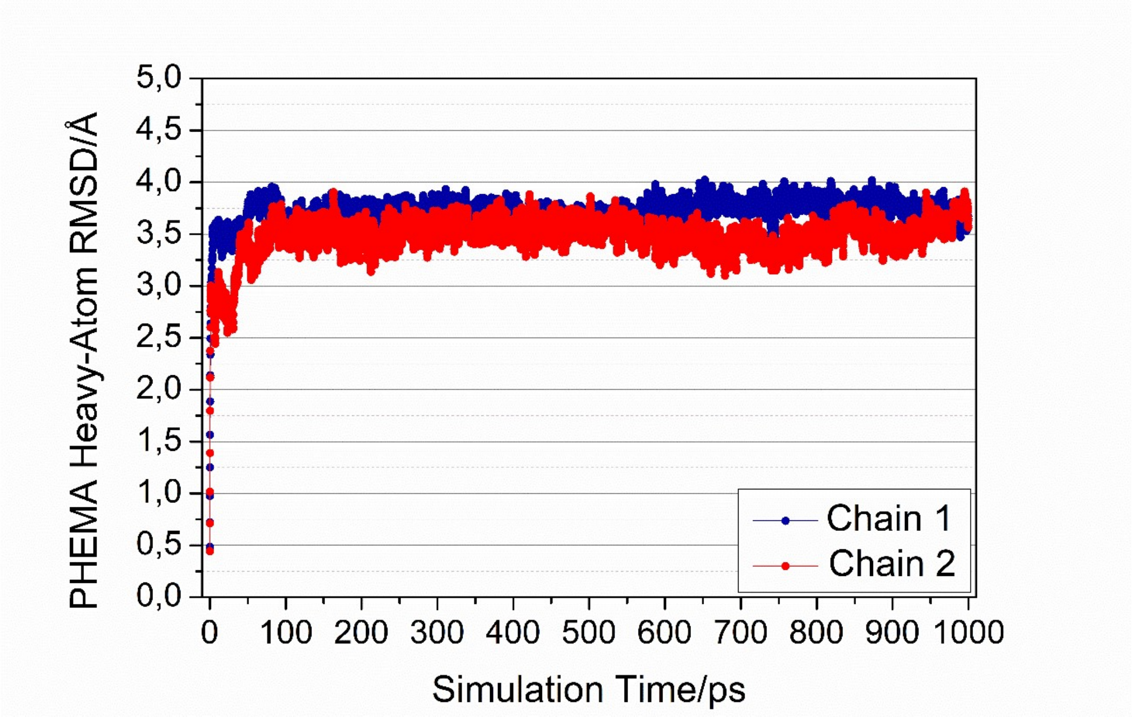

Fig. S5 Root mean square deviation (RMSD, in Å) over heavy-atoms from PHEMA chains for the "hydrophobic" PHEMA-GSNO 1 ns simulation.

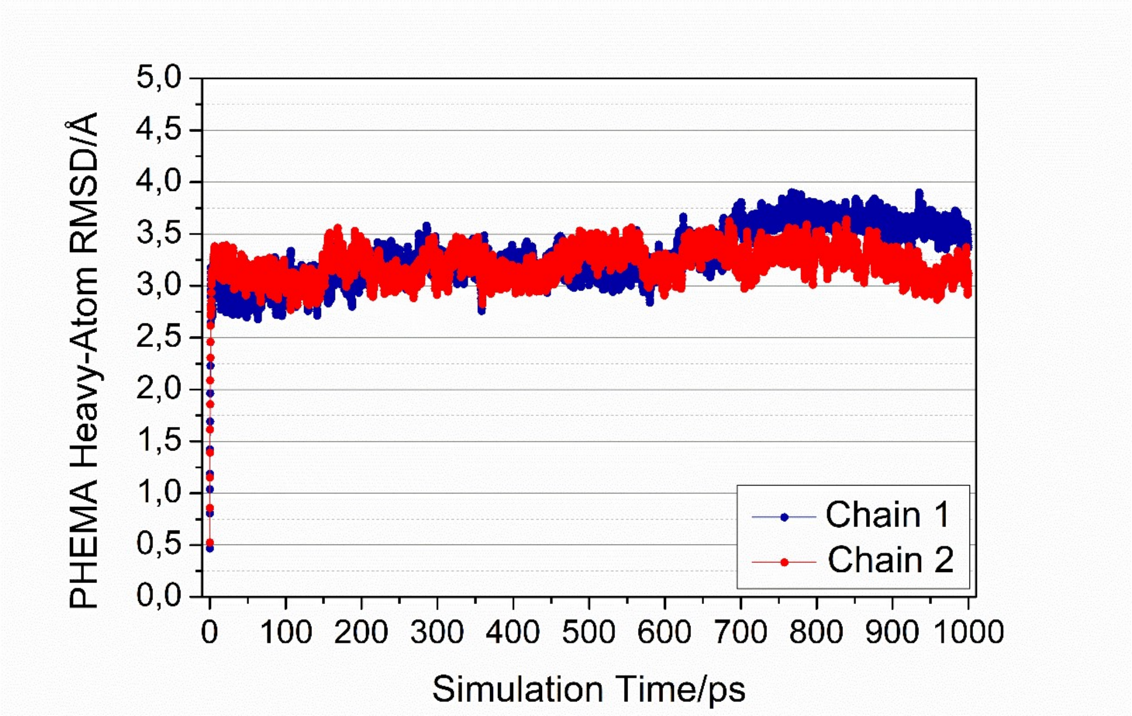

Fig. S6 Root mean square deviation (RMSD, in Å) over heavy-atoms from PHEMA chains for the "hydrophilic" PHEMA-SNAP 1 ns simulation.

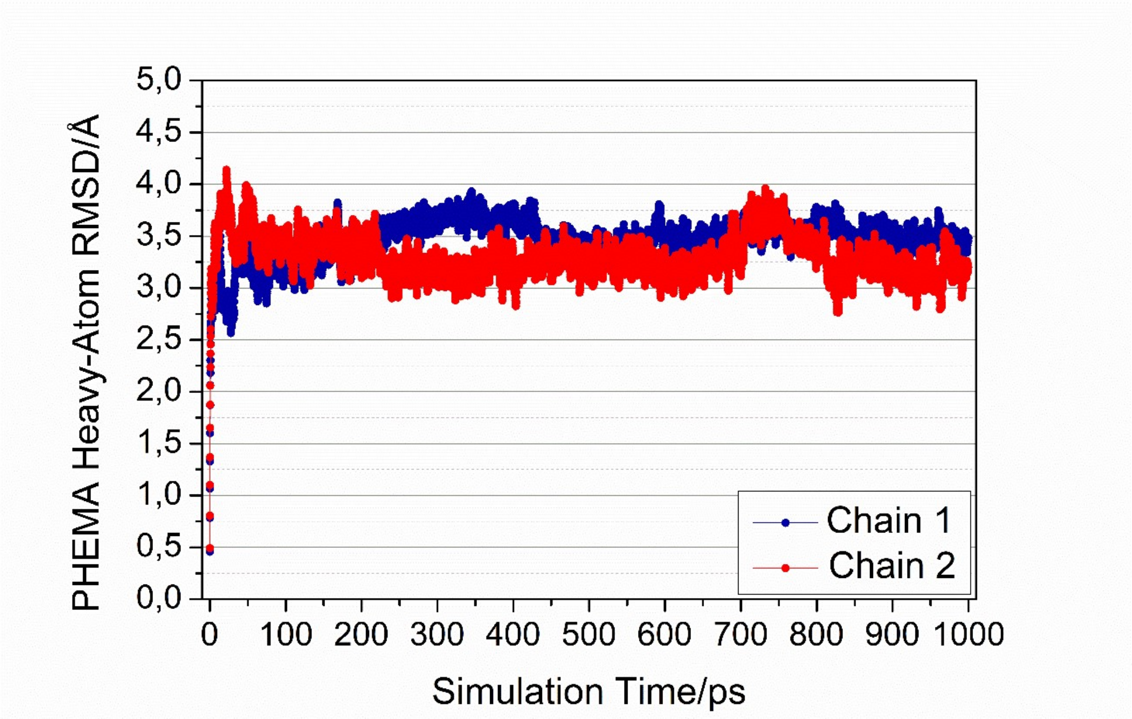

Fig. S7 Root mean square deviation (RMSD, in Å) over heavy-atoms from PHEMA chains for the "hydrophobic" PHEMA-SNAP 1 ns simulation.

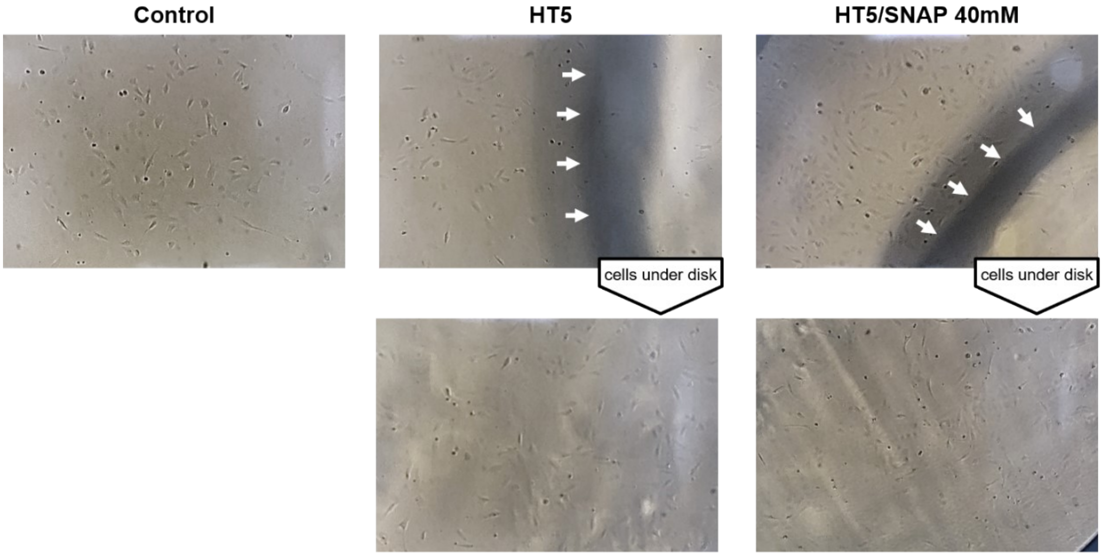

Fig. S8 Representative images of HUVECs cultured for 24 h at the bottom of a 96-well plate in the presence of HT5 disks with or without 40 mM SNAP, or without disks (control). In the upper panels, arrows indicate the position of the HT5 disk; lower panels show the corresponding cell layer beneath the disk at the same magnification.
